# Supplementary figures and images for: Sequence deeper without sequencing more: Bayesian resolution of ambiguously mapped reads
Source: PLoS Comput Biol. 2021 Apr 19;17(4):e1008926. doi: 10.1371/journal.pcbi.1008926 (PMC8084338; doi:10.1371/journal.pcbi.1008926)

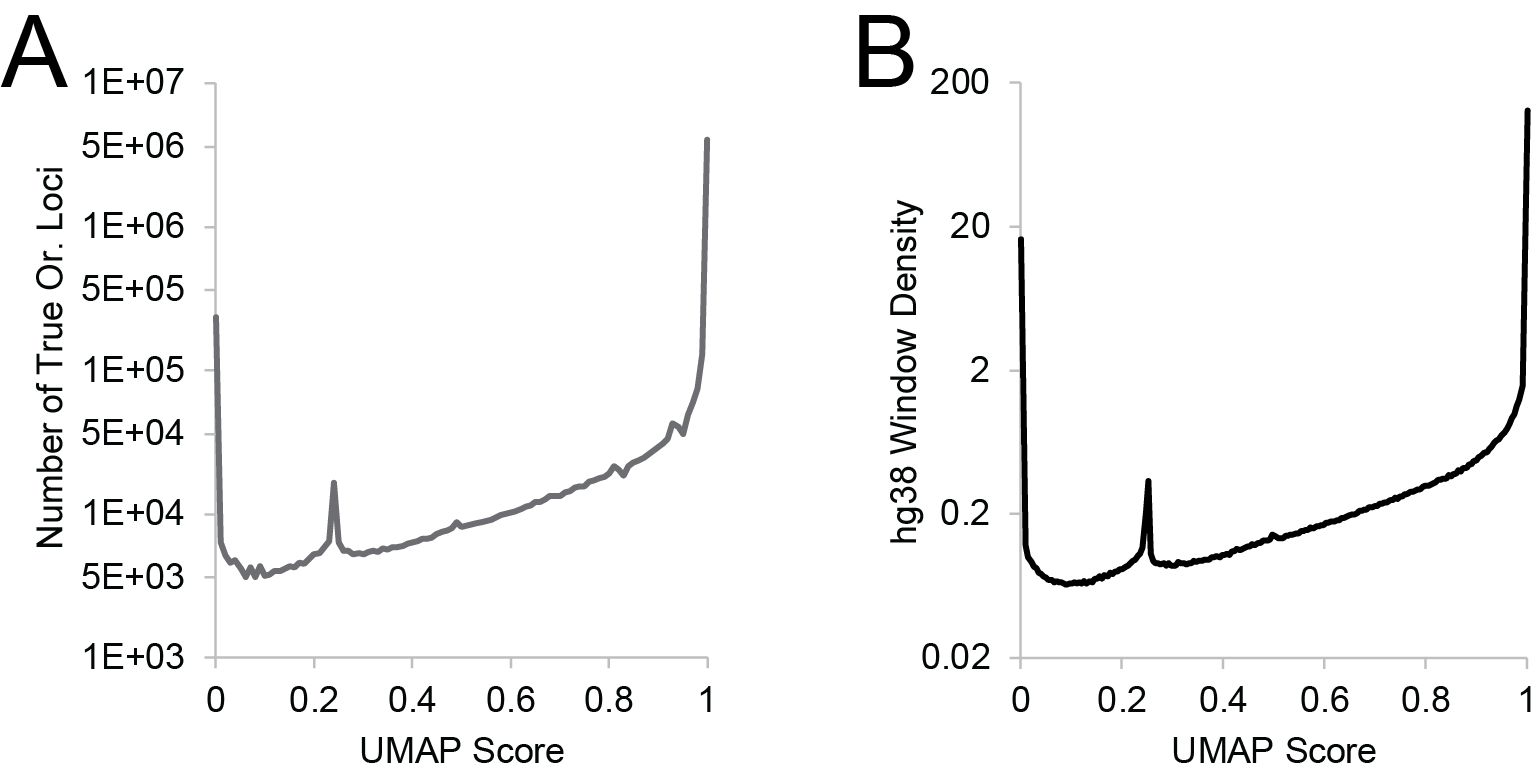

Supplement: S1 Fig — (A) Number of regions from the true origin loci vs. average mappability (UMAP50) score of the loci. (B) Density of UMAP50 scores of 200bp windows across the human genome (hg38). (TIF) [file pcbi.1008926.s001.tif]

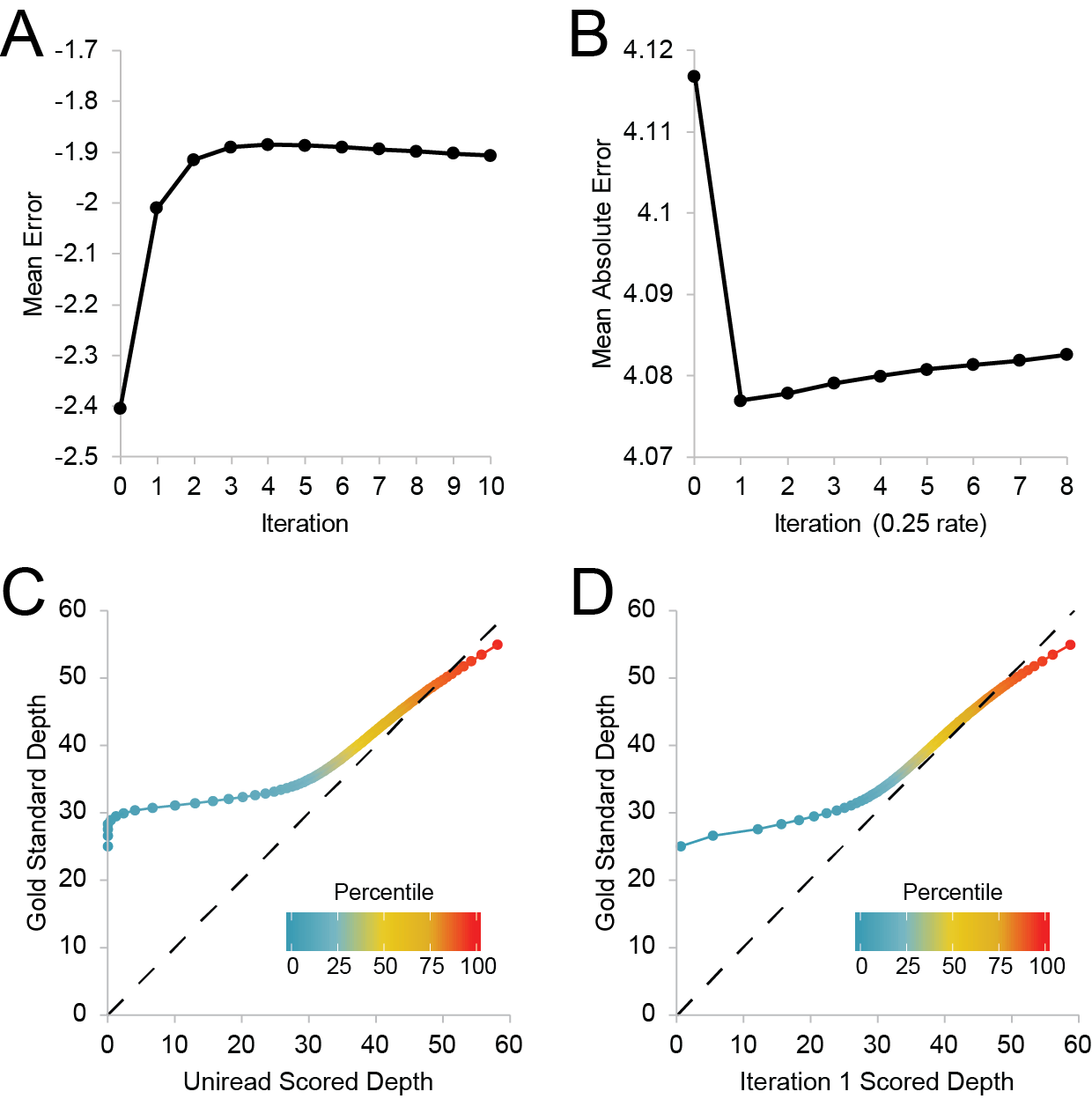

Supplement: S2 Fig — (A) Mean error of read depth at true origin loci in SmartMap scored mode vs. number of reweighting iterations. (B) Mean absolute error of read depth at true origin loci in SmartMap scored mode with a reweighting rate of 0.25 vs. number of reweighting iterations. (C, D) QQ plots of read depth in Gold Standard dataset vs. (C) uniread or (D) SmartMap (1 iteration) scored datasets. Color scale represents percentile of each point, from 1st to 99th percentiles. Dashed line represents line with slope of unity. (TIF) [file pcbi.1008926.s002.tif]

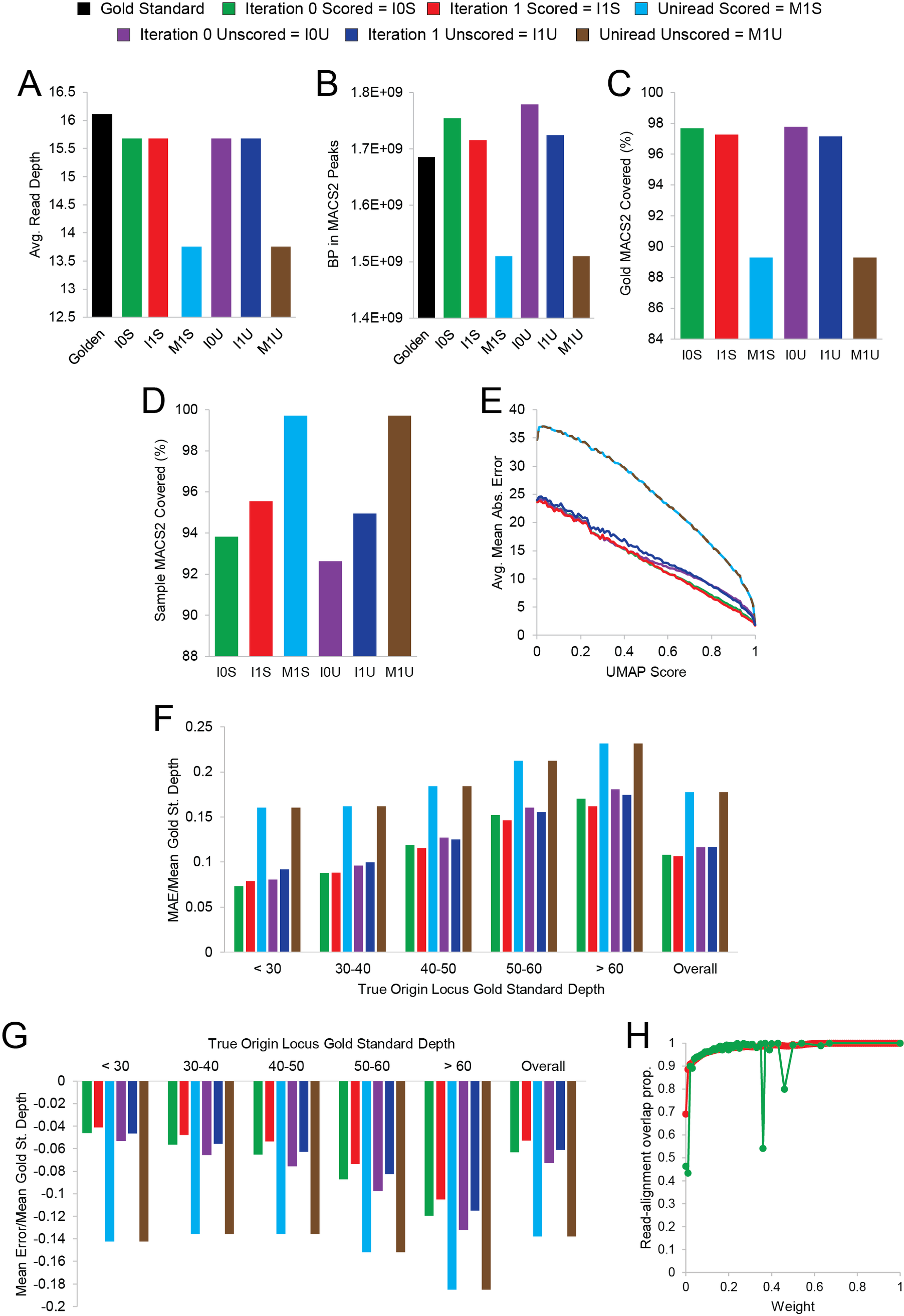

Supplement: S3 Fig — (A) Average read depth of each dataset genome-wide. (B) Base pairs covered by MACS2 called peaks for each dataset. (C) Percentage of MACS2 peaks in the Gold Standard dataset intersecting with MACS2 peaks in each other analysis, as percentage of base pairs covered. (D) Percentage of MACS2 peaks in each analysis intersecting with MACS2 peaks in the Gold Standard dataset, as percentage of base pairs covered. (E) Average mean absolute error vs. mappability score (UMAP50) of each dataset. Dashed lines are presented for readability of overlapping curves rather than discontinuities in data. (F) Mean absolute error of read depth at true origin loci for each dataset, with Gold Standard as the reference point, stratified by average Gold Standard read depth at true origin locus. (G) Mean error of read depth at true origin loci for each dataset, with Gold Standard as the reference point, stratified by average Gold Standard read depth at true origin locus. (H) Mean unweighted overlap proportion between alignment and true read origin as a function of alignment weight for the no-iteration (green) and one-iteration (red) scored SmartMap analyses. Overlap proportion is computed as a geometric mean of the proportion of the alignment and of the true read origin that overlaps with the other. (TIF) [file pcbi.1008926.s003.tif]

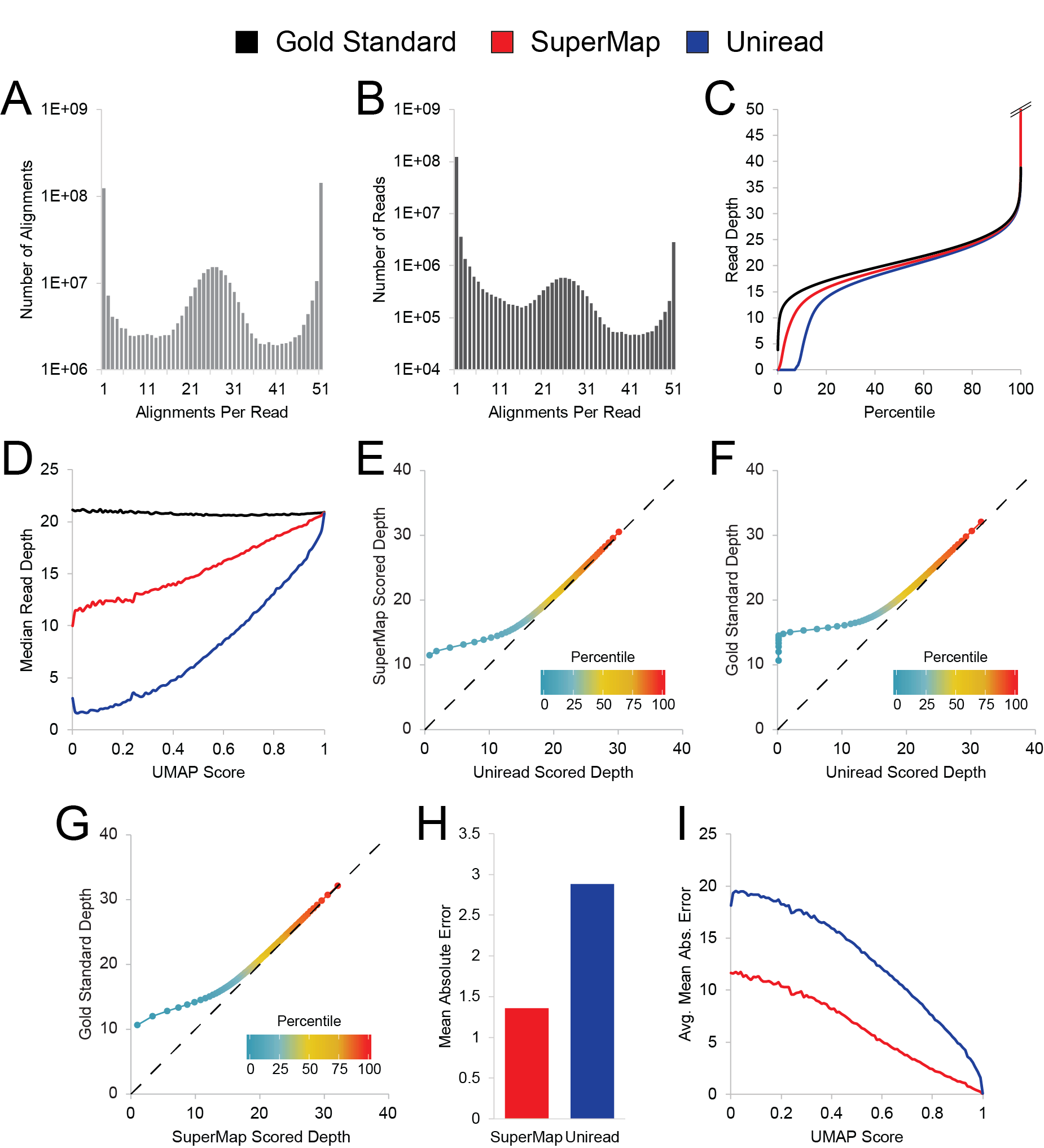

Supplement: S4 Fig — (A, B) Number of (A) alignments or (B) reads vs. number of alignments per read. (C) Quantile plot of read depth at the true origin loci. (D) Median read depth vs. mappability score (UMAP50) of the true origin loci. (E-G) QQ plot of read depth at true origin loci in the (E) SmartMap vs. uniread, (F) Gold Standard vs. uniread, and (G) Gold Standard vs. SmartMap scored datasets. Color scale represents percentile of each point, from 1st to 99th percentiles. (H) Mean absolute error of read depth at true origin loci for each dataset, with Gold Standard as the reference point. (I) Average mean absolute error vs. mappability score (UMAP50) of each dataset. (TIF) [file pcbi.1008926.s004.tif]

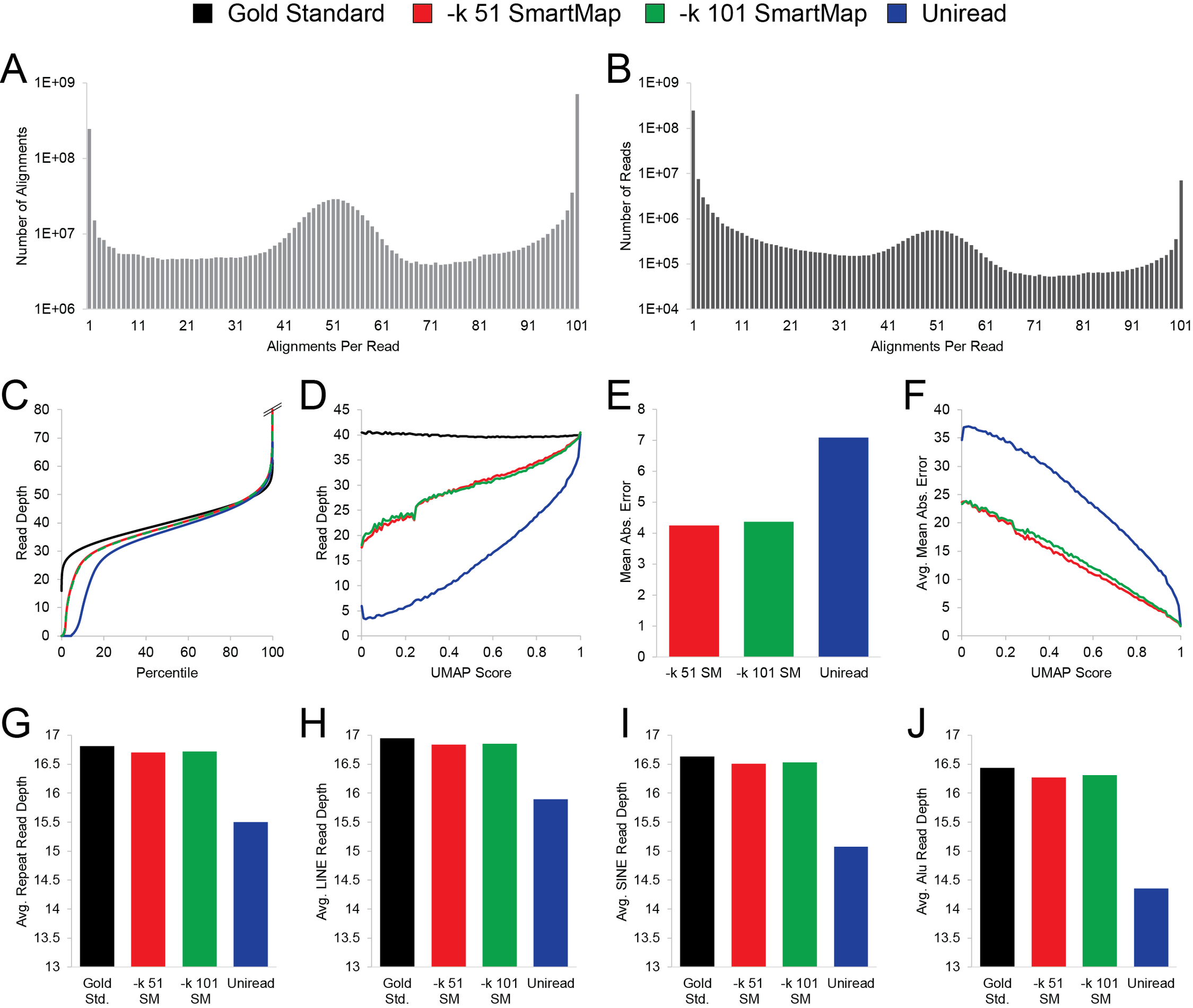

Supplement: S5 Fig — (A, B) Number of (A) alignments or (B) reads vs. number of alignments per read. (C) Quantile plot of read depth at the true origin loci. Dashed lines are presented for readability of overlapping curves rather than discontinuities in data. (D) Median read depth vs. mappability score (UMAP50) of the true origin loci. (E) Mean absolute error of read depth at true origin loci for each dataset, with Gold Standard as the reference point. (F) Average mean absolute error vs. mappability score (UMAP50) of each dataset. (G-J) Average read depth across the bodies of (G) all repetitive elements, (H) LINEs, (I) SINEs, and (J) Alu elements. (TIF) [file pcbi.1008926.s005.tif]

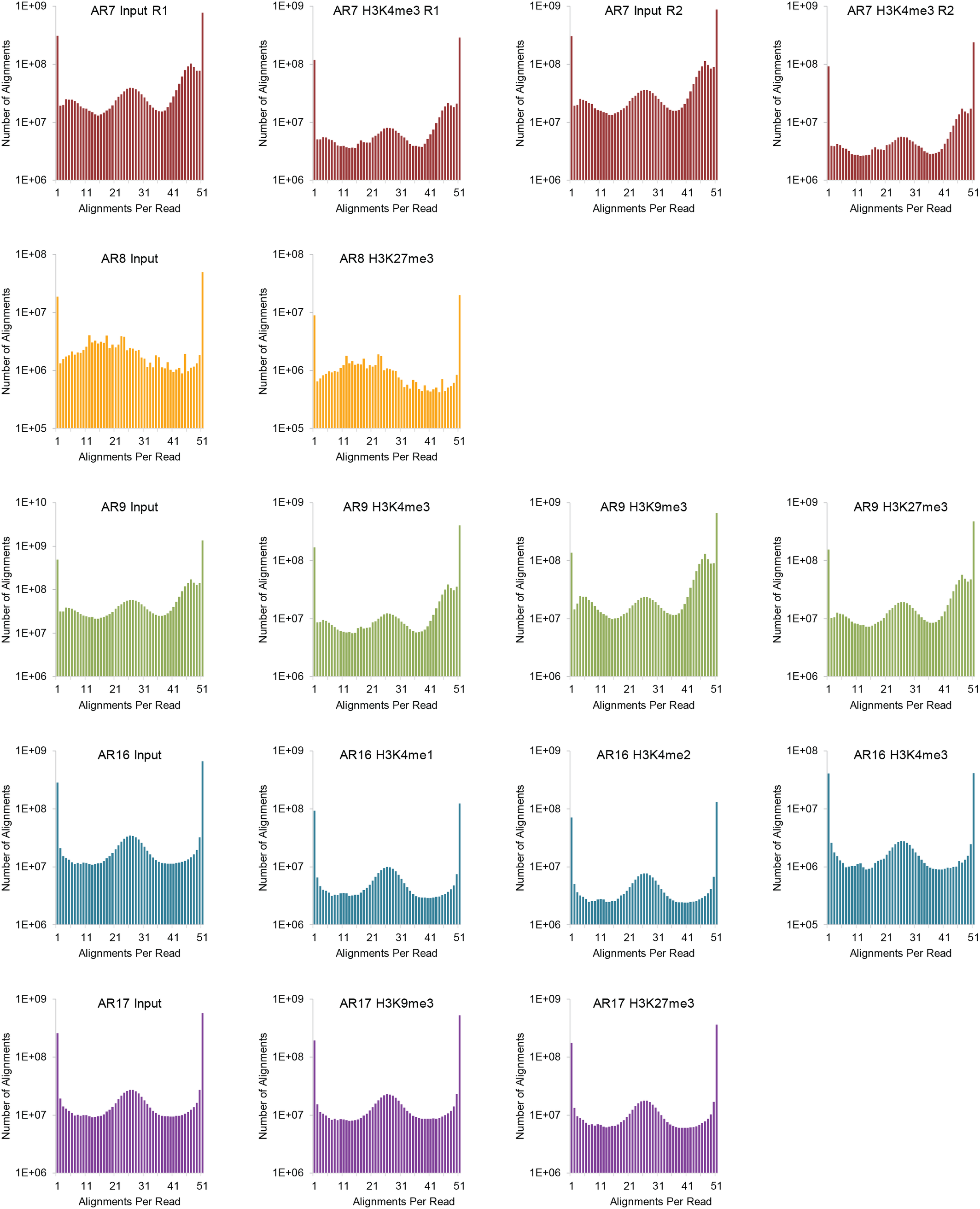

Supplement: S6 Fig — Number of alignments vs. alignments per read for each ICeChIP-seq dataset analyzed. (TIF) [file pcbi.1008926.s006.tif]

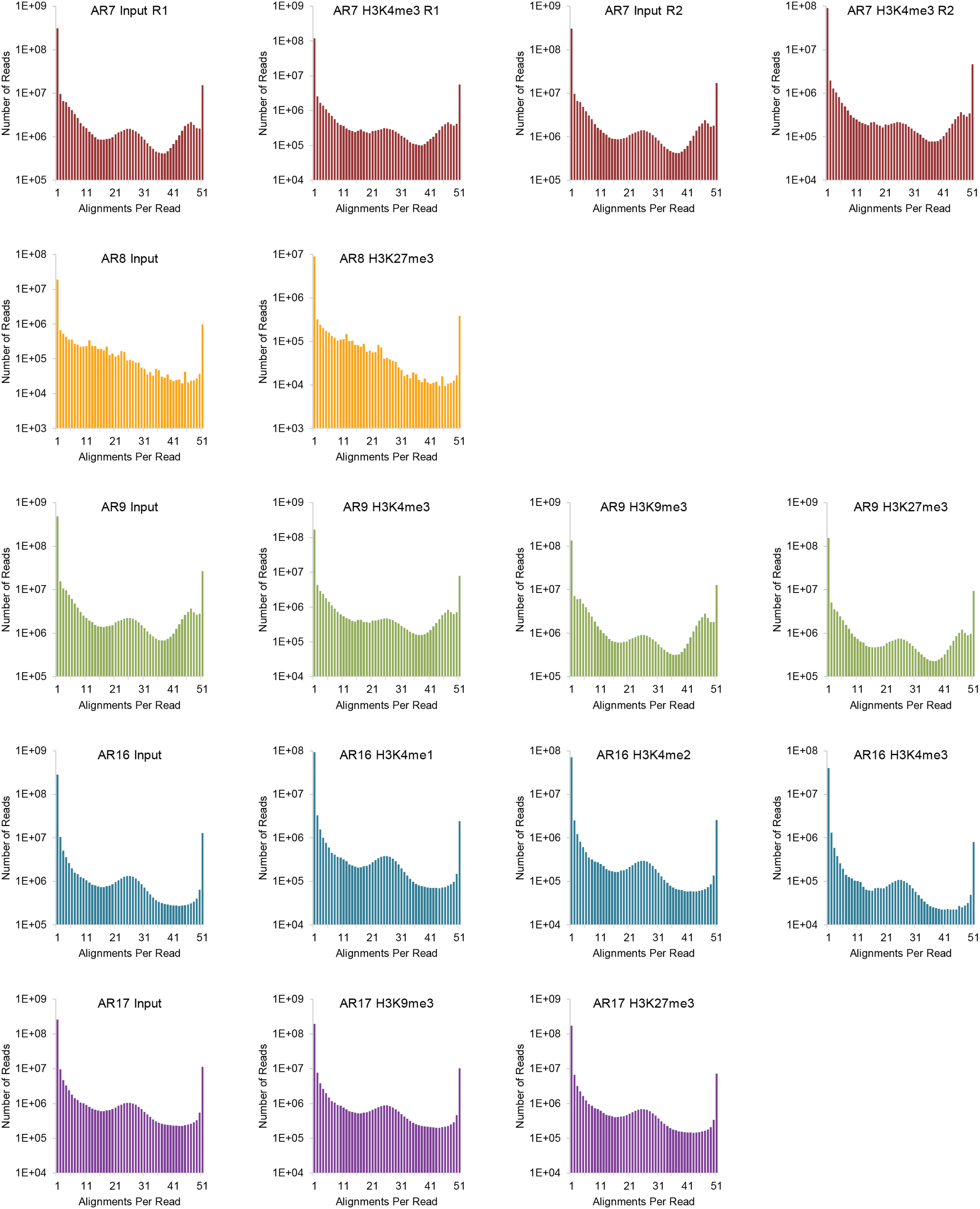

Supplement: S7 Fig — Number of reads vs. alignments per read for each ICeChP-seq dataset analyzed. (TIF) [file pcbi.1008926.s007.tif]

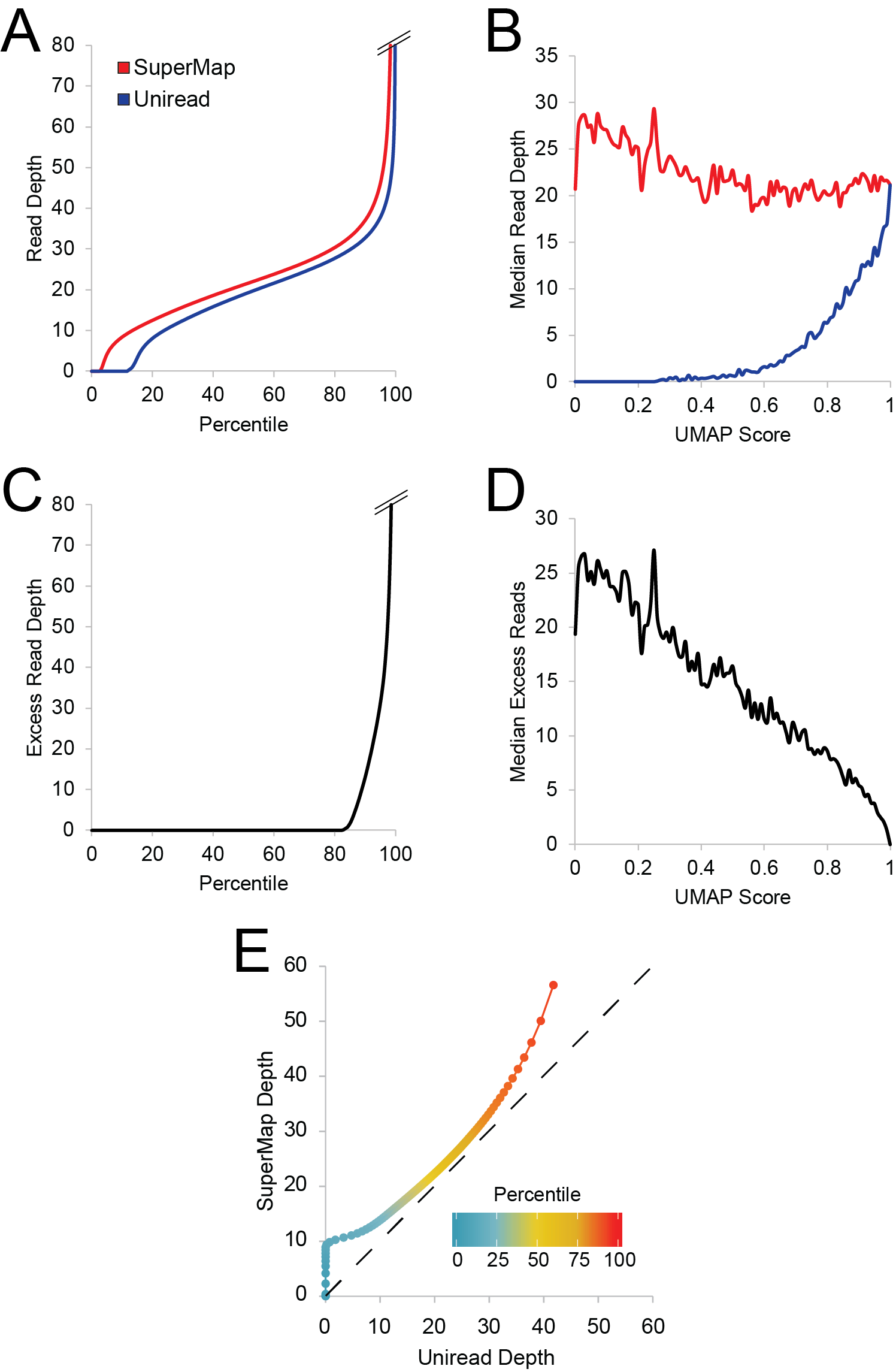

Supplement: S8 Fig — All analyses conducted on 200bp tiled genomic windows. (A) Quantile plot of read depth for SmartMap and uniread analyses. (B) Median read depth vs. mappability score (UMAP50) for SmartMap and uniread analyses. (C) Quantile plot of excess read depth in SmartMap relative to uniread analysis. (D) Median excess read depth vs. mappability score (UMAP50). (E) QQ plot of read depth in SmartMap vs. uniread analysis. Color scale represents percentile of each point, from 1st to 99th percentiles. Dashed line represents line with slope of unity. (TIF) [file pcbi.1008926.s008.tif]

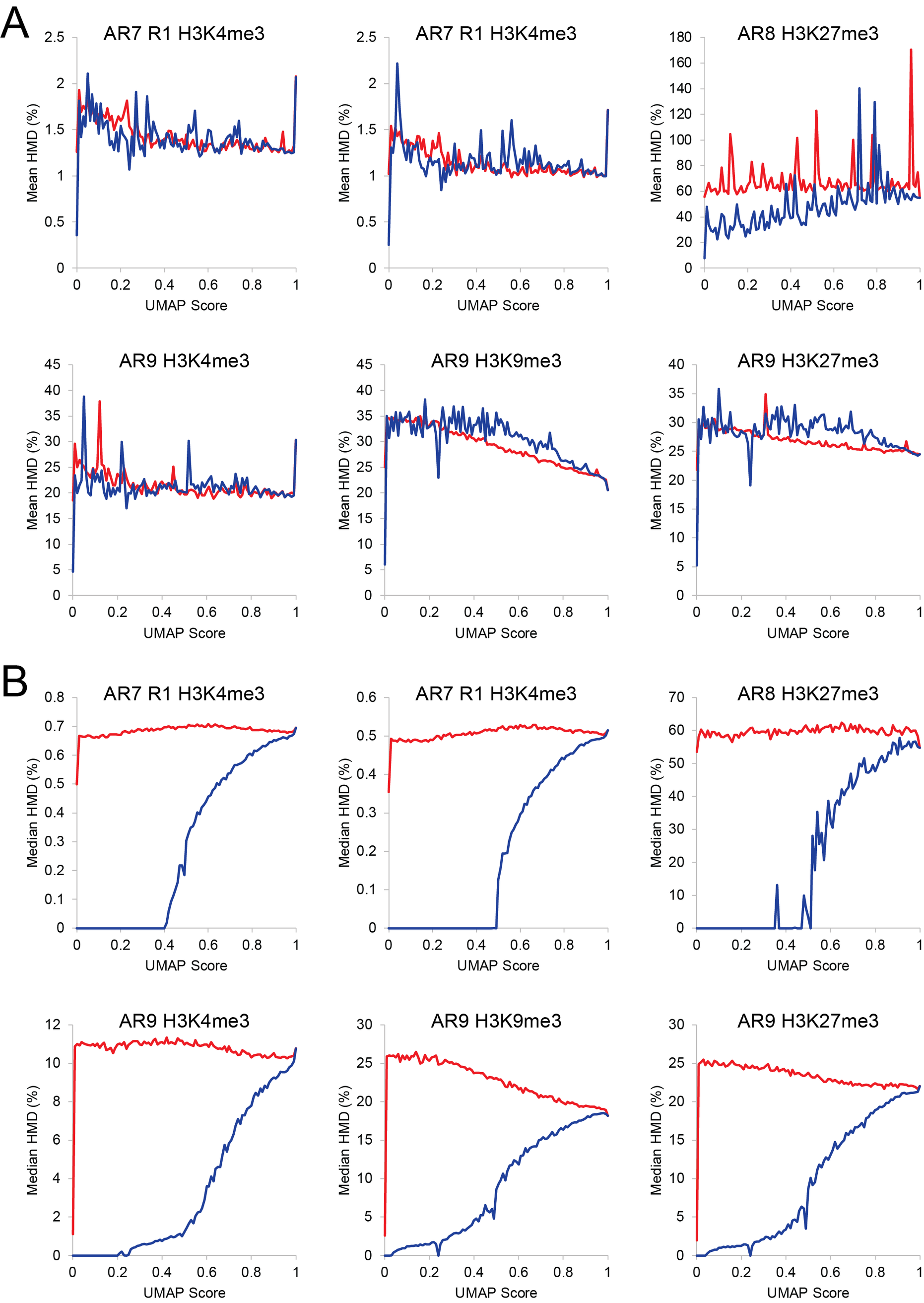

Supplement: S9 Fig — (A) Mean or (B) Median HMD vs. mappability score (UMAP50) for SmartMap and uniread analyses. Red line represents SmartMap analysis; blue line represents uniread analysis. (TIF) [file pcbi.1008926.s009.tif]

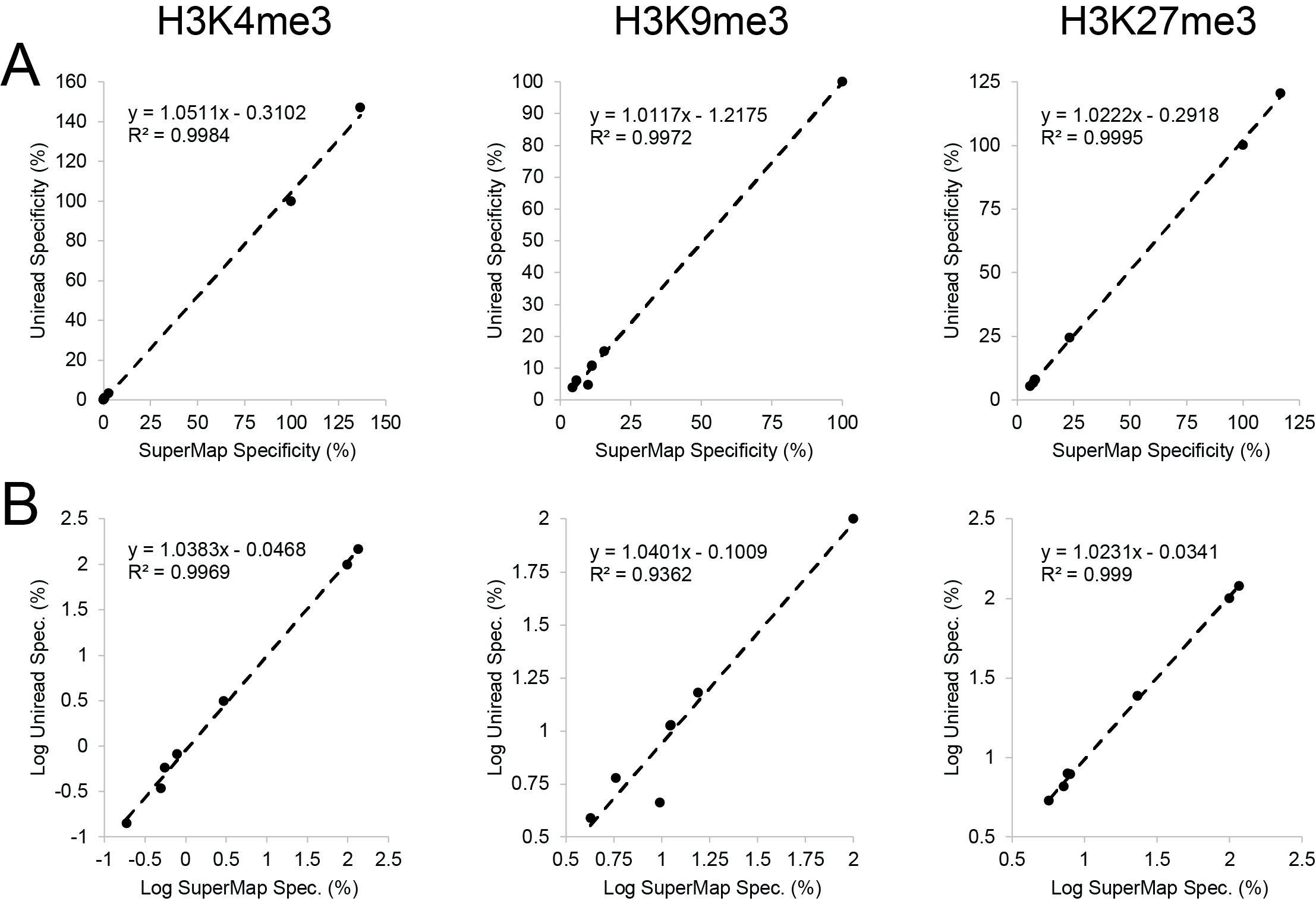

Supplement: S10 Fig — Scatterplots of (A) specificity or (B) log specificity for uniread vs. SmartMap analyses. Targets of pulldowns are H3K4me3 (left), H3K9me3 (centre), and H3K27me3 (right). Specificity is measured as the enrichment of each on- or off-target internal standard nucleosome as a percentage of on-target enrichment. (TIF) [file pcbi.1008926.s010.tif]

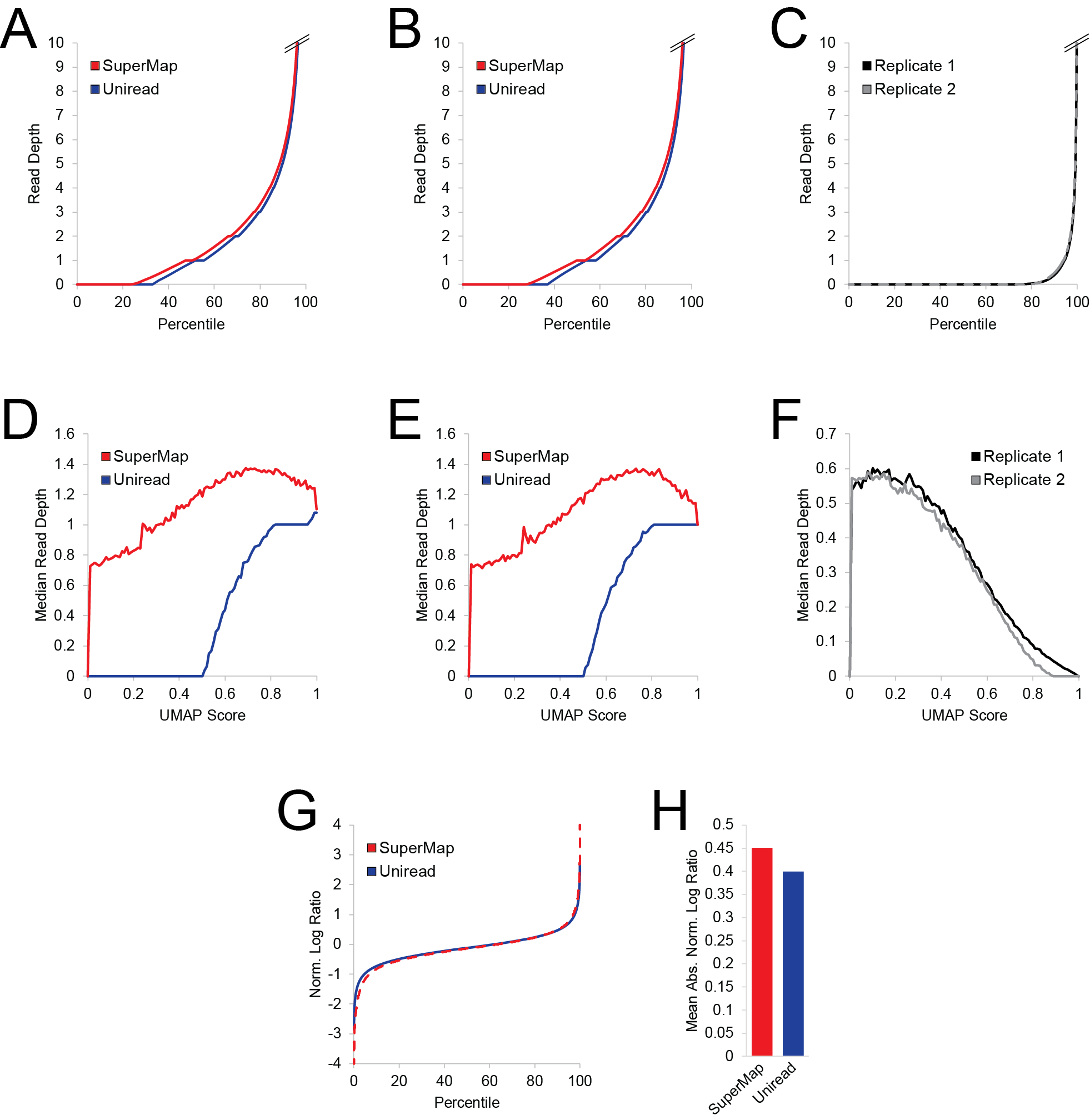

Supplement: S11 Fig — (A-B) Quantile plot of read depth at genomic windows in SmartMap and uniread analyses for (A) Replicate 1 or (B) Replicate 2. (C) Quantile plot of excess read depth in SmartMap datasets relative to corresponding uniread dataset for Replicates 1 and 2. (D-E) Median read depth vs. mappability score (UMAP50) in SmartMap and uniread analyses for (D) Replicate 1 or (E) Replicate 2. (F) Median excess read depth vs. mappability score (UMAP50). (G) Quantile plot of depth-normalized log ratio of read depth in Replicate 1 over Replicate 2, for SmartMap and uniread analyses. Graph breaks are present at both ends of the graph. (H) Mean absolute depth-normalized log ratio of the analyses shown in panel G. (TIF) [file pcbi.1008926.s011.tif]

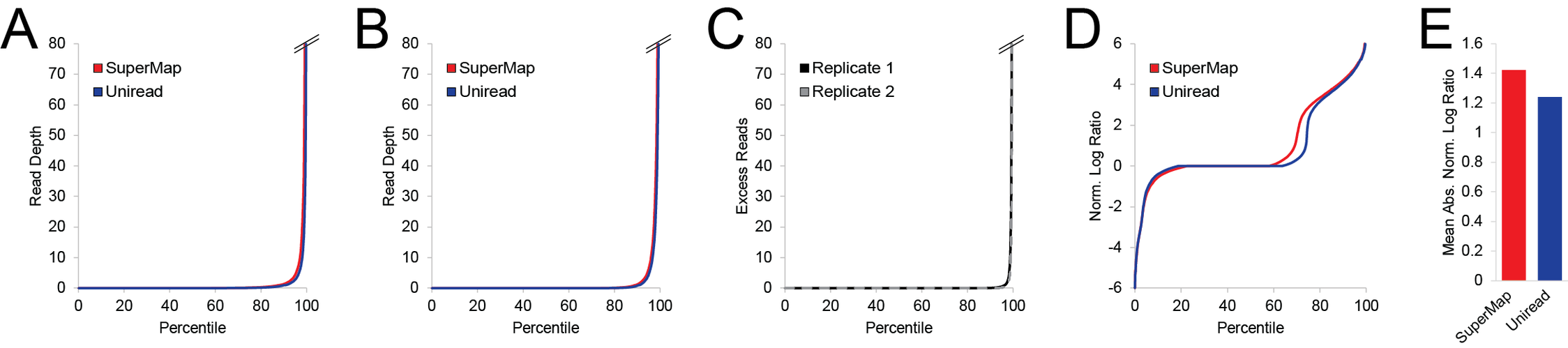

Supplement: S12 Fig — (A-B) Quantile plot of read depth at distinct Refseq genes in SmartMap and uniread analyses for (A) Replicate 1 or (B) Replicate 2. (C) Quantile plot of excess read depth in SmartMap datasets relative to corresponding uniread dataset for Replicates 1 and 2. (D) Quantile plot of depth-normalized distinct Refseq gene log ratio of read depth in Replicate 1 over Replicate 2, for SmartMap and uniread analyses. Pseudocount of 10−7 was added to each gene due to the high number of genes with zero read depth. Graph breaks are present at both ends of the graph. (E) Mean absolute depth-normalized log ratio of the analyses shown in panel D. (TIF) [file pcbi.1008926.s012.tif]

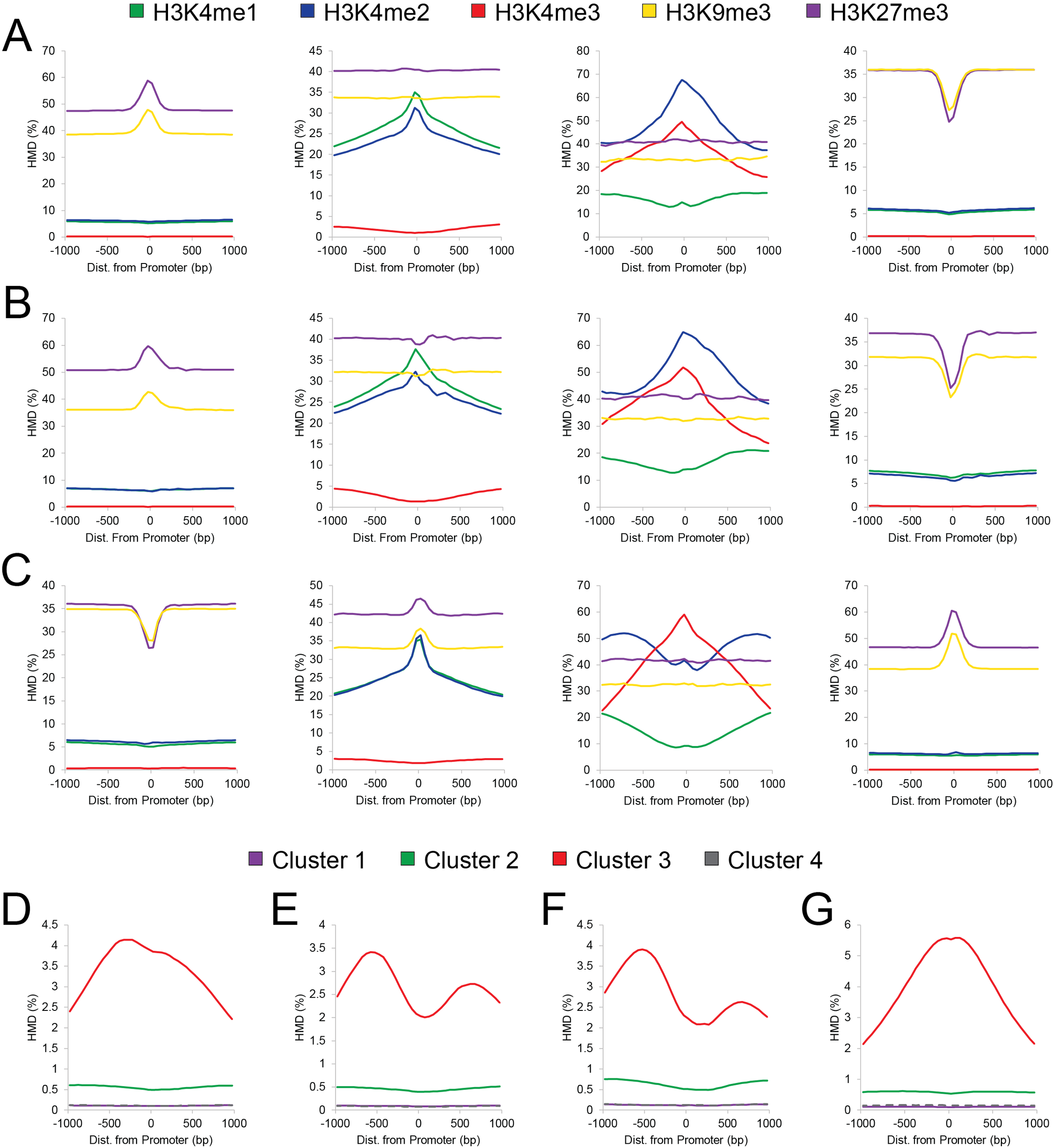

Supplement: S13 Fig — (A-C) HMDs of modifications about promoters of (A) LINEs, (B) SINEs, or (C) simple repeats separated by k-means clustering conducted on the appropriate set of repetitive elements. (D-G) Total ATAC-seq read depth across both replicates about promoters of (D) all repeats, (E) LINEs, (F) SINEs, or (G) simple repeats. (TIF) [file pcbi.1008926.s013.tif]

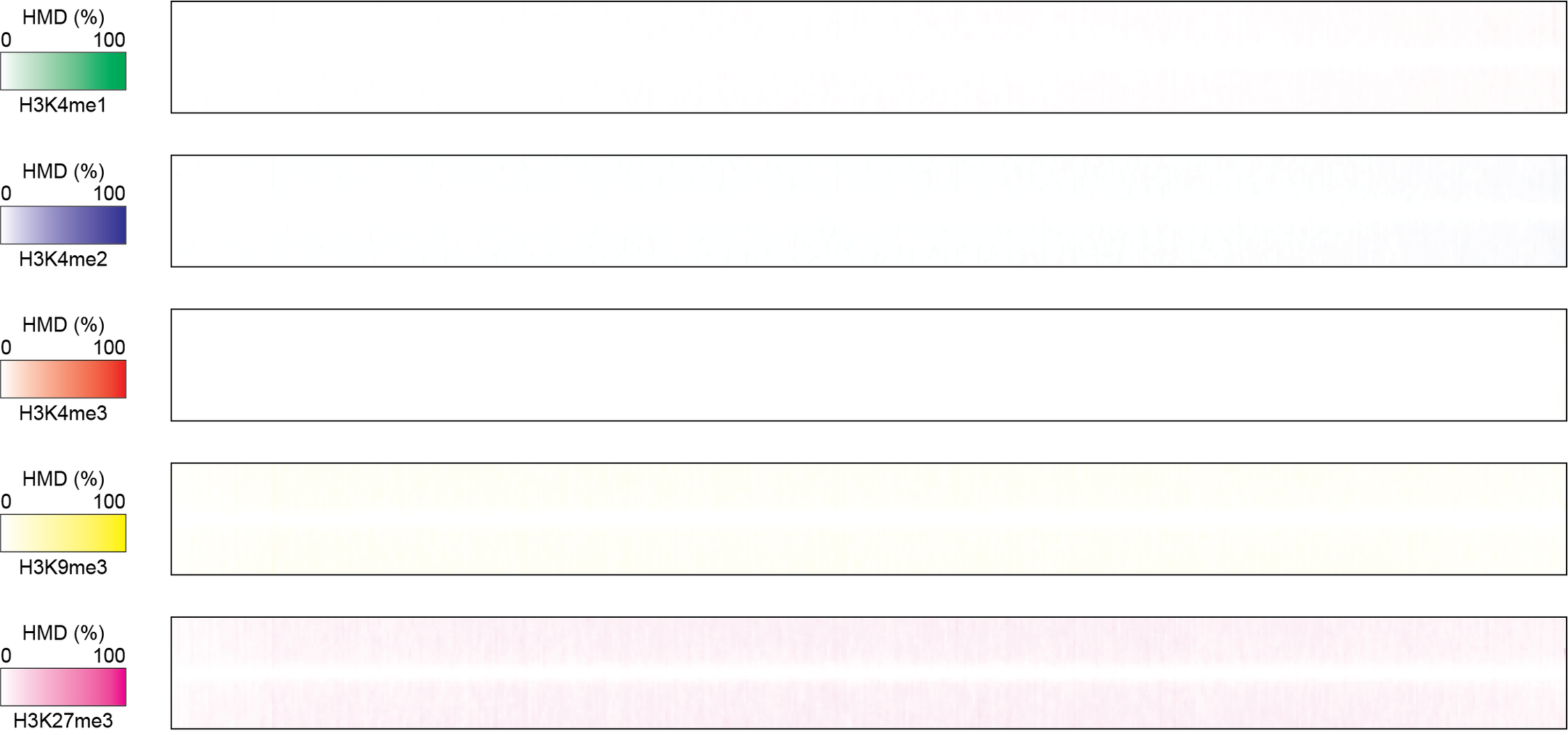

Supplement: S14 Fig — Heatmap of repeat promoters with measurable nonzero HMD only in SmartMap analysis, sorted on first principal component of repetitive elements. (TIF) [file pcbi.1008926.s014.tif]
