# Supplementary material for: Sequence deeper without sequencing more: Bayesian resolution of ambiguously mapped reads
Source: PLoS Comput Biol. 2021 Apr 19;17(4):e1008926. doi: 10.1371/journal.pcbi.1008926 (PMC8084338; doi:10.1371/journal.pcbi.1008926)
Supplement: Attachment — Submitted filename: Shah and Ruthenburg Response to Reviewer Comments.docx [file pcbi.1008926.s015.docx]

**Response to Reviewer Comments**

First, we would like to thank the reviewers for taking the time to review our manuscript and offer us suggestions on how to improve this work. It is clear to us that all the reviewers engaged closely with the material, and we are sincerely appreciative of your very helpful comments. Inspired by your comments, we have made many changes and additions to our algorithm and our manuscript that we feel vastly improves this work.

Below, we have written a response to each of the points made. All references to line numbers refer to the clean document, as the line numbers were changed in accepting all tracked changes.

**Reviewer #1: The authors present MultiMap, a method and software tool for aligning and reallocating multimapping reads. The core method uses bowtie2 set to report up to 51 alignments and then applies either a weighted (according to MAPQ) or unweighted EM procedure for a single iteration.

The C++ software implementing MultiMap is open source and available at:** [**https://github.com/shah-rohan/multimap**](https://urldefense.com/v3/__https:/github.com/shah-rohan/multimap__;!!OaZRxjSJGsACmqMyc8s!cZ4N_hV_mPszW2YB_wSn3gRi8C69UpQEciKym27-oWyCBw9TRcHUg0WMOG-DybHBHEC9v7E$)**. I was able to build it with a minor hiccup (described in minor comments below).

The idea is far from new; there have been papers about this topic and suggesting substantially the same solution going back about 10 years (e.g.** [**https://projecteuclid.org/euclid.cis/1268143264**](https://urldefense.com/v3/__https:/projecteuclid.org/euclid.cis/1268143264__;!!OaZRxjSJGsACmqMyc8s!cZ4N_hV_mPszW2YB_wSn3gRi8C69UpQEciKym27-oWyCBw9TRcHUg0WMOG-DybHBzGYS1D0$)**). That said, the authors do seem to be addressing a presently unmet need for methods that (a) handle-paired end reads, (b) are able to choose alignments for passing downstream, which is more widely applicable than quantifying a set of intervals, (c) handle strandedness. Thus, this method seems to be much more generic and likely to aid a variety of downstream tools than any other I know of.

Having said that the overall idea is not new, the idea of applying Fenwick trees to the bookkeeping is new and interesting. I am a little surprised this wasn't highlighted more as it's a good idea and I don't recall seeing it in any of the other papers describing a multimapper method.**

We sincerely appreciate this comment. However, we feel compelled to note that we cannot take credit for the idea of using Fenwick trees in multimapping methods; in 2011, the Keleş group published a method (CSEM) that used Fenwick trees to store read information (Chung et al., *PLOS Comp Biol* 2011). To be sure, our application of Fenwick trees expands upon the previously published method by allowing for extension to paired end and stranded data, but the broader concept is not entirely ours. To make this clearer to the reader, we have explicitly made note of this on lines 168-171. Nonetheless, we are glad to hear that you feel our application is of interest!

**The strongest point of the paper are the experiments, which are extensive and cover multiple downstream applications of multimapper reassignment.**

**Major comments

1.1) The authors could do more to investigate exactly why multimapper assignment performance suffers after the first iteration of reassignment. It should be easy to check the simulation results to see whether this is primarily because "real" peaks are pulling in reads not belonging to those peaks, or if it's for another reason.**

This is a great point and also one that Reviewer #3 raised (Major Comment 3.6). To look at the reason why performance suffers after the first iteration, we have conducted two major analyses, now discussed in lines 230-265 of the manuscript.

First, as you suggest, we wanted to determine whether the “real” peaks are pulling in reads not belonging to those peaks. In essence, we think of this question as: is the increase in error systematic (e.g. real peaks tend to “pull in” too many reads or, alternatively, “lose” real reads systematically), or is this increase in error random (i.e. real peaks gain and lose reads roughly equally)? To assess this, rather than computing mean *absolute* error at true origin loci for each iteration, we computed the mean error. This would allow us to detect whether mean absolute error increases at each iteration were systematic (with a mean error not equal to zero) or random (with a mean error roughly equal to zero). What we find is that as iterations increase, the mean error becomes slightly less negative, suggesting that over time, the true origin loci are “pulling in” more reads (Fig S2A). However, the mean error is still negative, and the change from iteration 1 is small and doesn’t change markedly from 2-10 iterations. As such, we interpret that there is not a systematic “pulling in” over increasing numbers of iterations, suggesting that the error is largely random and non-systematic.

Our second analysis to this end was to develop a new mode for MultiMap reweighting that we refer to as “slow reweighting,” as discussed in a response to Reviewer #3’s sixth comment. In the interests of reducing repetitiveness, we will summarize here, but further details are present in the response to Reviewer #3 if you are interested in how we implemented this analysis. Briefly, this new method introduced a rate parameter *r* such that, rather than fully reweighting an alignment on each iteration (when *r* = 1), we could instead only partially reweight each alignment on each iteration (when *r* < 1), allowing for slower reweighting and, hopefully, preventing “overshoots” of reweighting that would compound in error with further iterations.

With this new method, we found that the mean absolute error was much more stable with increasing numbers of iterations (Fig S2B). This suggested to us that standard MultiMap analysis after one iteration could “overshoot” the reweighting at many loci, which would then compound and pull in more and more (or fewer and fewer, depending on the direction of overshoot) reads with more reweighting cycles. By contrast, with slower reweighting, this overshoot could be better managed.

As such, we feel that these two analyses indicate to us that the increase in error is not systematic “pulling in” of incorrect reads across all true origin loci but, rather, represents relatively random “overshoot” of the correct weights after one iteration that then compound in error with further reweighting cycles. With this knowledge, our new rate parameter and “slow reweighting” mode of MultiMap can enable the end user to better tune their analyses.

Thank you for the suggestion and for prompting us to study this more closely – we feel your suggestions and these analyses have helped us improve and expand our method!

**1.2) There should be some discussion of the difference in computational efficiency between the uniread approach and MultiMap. Asking bowtie2 to report up to 51 alignments per read must have some measurable effect and it is important for readers to understand what it is.**

**Related to previous point: the paper should measure how much time and space overhead are added by the need to populate and query the Fenwick tree.**

These are both very good points. To this end, we have now changed Table 8 to outline the time and memory required for each step in a more detailed manner for the simulated dataset, as compared to uniread and BM-Map analyses (to the extent that the latter functions at all). (Consistent with Reviewer #3’s comments, we have not included the RNA-seq benchmarking because we are working to de-emphasize the use of MultiMap in RNA-seq, as that is not the best use case for this software, as echoed in the introduction to Reviewer #3’s comments.) Thank you for this suggestion!

**Minor comments**

**1.3) I recommend that the authors keep only source files and scripts in the repo and not pre-built libraries (.a files) or object files (.o files). I had to remove the .a and .o files from the gzstream subdirectory before I could successfully build the tool on my Mac.**

Thank you! We really appreciate this level of fine-grained assessment, and we sincerely appreciate the help – this will make it much easier for the end-users to use the software. We have removed these libraries and object files and have updated our makefile recipe such that it can be run from the main project directory to build both gzstream and MultiMap. **1.4) The manuscript should tell readers what the UMAP50 score is. As of now, there just seems to be a reference to a paper, but I don't think it is a widely understood concept.**

This is a good idea – we have clarified this concept with an explanation of what the UMAP50 and an example calculation in the main text at the point when we first introduce the concept. This is now explained on lines 182-190.

**1.5) A very minor point: it is typical to say "Gold Standard" rather than "golden standard"**

Thank you – we have changed this throughout the manuscript and figures!

**Reviewer #2: The authors present a new approach to handling multi-mapped reads, in particular for depth based analyses like ChIP-seq and RNA-seq. The approach works by reweighting MAPQ scores with all alternative mappings of a read and then iteratively reweighting MAPQ scores via the averaged weights of all reads mapping to the same locus.


\# Major comments

2.1) From reading the manuscript, I am not entirely convinced that the approach to give reads at high coverage loci a bigger weight is correct in any case. I can imagine that there are loci which (wrongly) attract a lot of reads from elsewhere in the genome. With this approach, it seems to me that reads mapping at such loci will get an artificially good weight, thereby even increasing the systematic error that the read mapper made there. How does the method protect against such a scenario, or alternative, why is it likely to be not a problem?**

This is an eminently reasonable concern. You’re right that there are certainly going to be some set of regions that wrongfully attract a lot of reads; this is part of the basis for defining some regions of the genome as “high signal regions” in datasets such as the ENCODE blacklist, and it’s likely that there are regions of the genome that are similarly problematic without being listed in the blacklist. As such, those regions will likely receive an artificially high weight and have an inflated error.

The reason we are not too concerned about that point is because of the proportion of the genome that these regions represent.

To assess for this, we looked at the average read depth of 200bp genomic windows across the genome for our simulated datasets (which include off-target genomic reads, as described in the response to your fifth major comment). The maximum of these read depths for the Gold Standard dataset was roughly 83, which served as a rough marker for our definition of a “high signal region” in each of the other datasets; we defined a genomic window as having high signal if it had an average weight of greater than 60, 70, 80, or 90 reads (in separate analyses). Notably, the Gold Standard was generated directly by simulation and was not subjected to alignment and mapping, meaning that it should not contain artifactual high-signal regions as the result of improper sequencing or alignment.

We then computed the number of genomic regions with greater than 60, 70, 80, or 90 reads in each of the Iteration 0 (MultiMap), Iteration 1 (MultiMap) and Uniread datasets in scored mode to assess for how many of these roughly-defined “high signal regions” were present in each of these datasets. These results, both in absolute numbers and as a proportion of the genome, are outlined in Table 3. We found that even using the relatively lax threshold of 60 reads, less than 0.3% of the genome would be classified as a high-signal region with MultiMap using 1 iteration – nearly comparable to that of the Gold Standard. Using a higher threshold such as 80 reads, only 0.04% of the genome had such a problem.

To summarize, we absolutely agree that this is a valid concern and, indeed, represents an ongoing problem with mapping NGS reads in general. Fortunately, the proportion of the genome for which this is a major problem with 1 iteration of reweighting appears to be quite small; at worst, more than 99.7% of the genome still does not appear to have this high-signal problem.

Nonetheless, this is still an important drawback of MultiMap that the end user should be aware of, and to that end, we have included explicit discussion of this point on lines 441-462.

**2.2) The manuscript lacks a comparison against the other cited methods. I understood that they do less and CSEM fails, but the others could still be applied in order to illustrate how MultiMap improves over them.**

This is a good point – to this end, we have explicitly implemented the uniread method and also implemented the random alignment selection methods. The uniread method is the most common analysis for NGS data. Random alignment selection is also frequently used for multiread analyses. As we noted briefly in the discussion, the true “random selection” of alignments essentially corresponds to the MultiMap Iteration 0 dataset, as the weights described there are functionally the expected value of the set of randomly selected alignments, as the weights would be equal to the probability of selection. However, for the sake of completeness, we have also explicitly run a random alignment selection analysis on the multiread alignments. These are explicitly compared against each other by mean absolute error in Table 8, where we find that the MultiMap analysis with one reweighting cycle outperforms the no-reweighting MultiMap (Iteration 0), random alignment selection, and uniread analyses.

We attempted to implement the BM-Map software as well. Unlike CSEM, this software was able to at least read the alignments into the software (albeit with extremely high memory requirements and considerable CPU time). However, after reading the alignments, the software returned an error and crashed with no results outputted. It is possible that there were problems with the formatting of the SAM file input that caused the software to break; the authors of BM-Map themselves note that the tool has very stringent input file requirements. Unfortunately, these requirements were not clearly delineated, so we are at a loss for how to fix them – or even if the input file is the problem. This problem was observed both with the precompiled binary as well as our compilation of the source code.

We have reached out to the authors of the CSEM and the BM-Map software to try to get their tools running on our systems. The corresponding author of BM-Map wrote back and informed us that, due to its age, they no longer offer technical support for the software. We have not yet heard back from the corresponding author of CSEM. If we are able to get their software to run with their assistance prior to publication, we will add these benchmark comparisons to the manuscript as well.

The other cited methods are not particularly applicable for our implementation/use case and, as such, have not been included in this analysis. First, several of them are primarily designed for RNA-seq analysis and quantification of pre-defined features rather than global analysis of “peak type” NGS data (as Reviewer #3 put it). Especially because, at Reviewer #3’s suggestion, we are working to de-emphasize the role of MultiMap for analysis of RNA-seq data, we feel this would not be a useful (or feasible) comparison point for our fundamentally different datasets.

Amongst the remaining methods, we attempted comparison to CSEM and BM-Map but, as noted, found that they did not function properly on our datasets. Perm-seq (PMID: 26484757) relies upon using DNase-seq as an informative prior to then refine ChIP-seq alignments, at which point it conducts peak calling and outputs called peaks; AREM (PMID: 22035330) also serves a similar function. While these are fascinating and useful methods, it’s not particularly similar to our method, which avoids using external datasets as priors. Indeed, it’s not even obvious to us how we would compare our simulated data (with the Gold Standard) and assess for error with this method. As such, because of this marked dissimilarity between the intended use of Perm-seq or AREM and MultiMap, we have not included them in the set of comparisons. The work by Liu et al. (PMID: 32019414) is similar to other methods (namely, random selection and reweighting alignments non-iteratively) but does not provide software, code, or sufficient algorithmic detail to reimplement their exact method, making direct comparison impossible.

**2.3) As I understood it, the reweighting of the reads could be used to provide basically improved MAPQ values (see Discussion), i.e. a better estimate of the probability that a read (pair) alignment is mapped to the wrong place. With the simulated golden standard, it should be easy to actually calculate whether the new updated MAPQ scores are correct, or at least better than the initial MAPQ scores from the mapper. Among all read (pairs) with an estimated MAPQ probability p, there should be only p wrongly mapped. The true fraction of wrongly mapped read pairs should be calculated for each p, and plotted against the values of p. If the algorithm works well, the result should be nearly a diagonal. Such an analysis would generate much more trust in the used weighting than the indirect approaches done here (although they are of course also very reasonable).**

This is a great idea! To enable this kind of analysis, we modified our MultiMap software to be compatible with outputting the reads and alignments with their associated weights. We then computed the proportion of correct mappings in two separate ways. First, we computed the proportion of alignments with a given weight overlapping with its corresponding Gold Standard read; this is presented in Figure 3I. Second, of the alignments that were overlapping with the corresponding Gold Standard read, we computed an overlap proportion between the alignment and Gold Standard read, then calculated the average overlap proportion at each alignment weight; this is presented in Figure 3J.

With this analysis, we found several interesting features. First, because of the way that alignment weight is computed; the highest possible weight for a multiread without any iteration cycles is 0.67 (two alignments, with one being of high quality and one being of low quality, with initial quality proportions of 1 and 0.5, respectively). As such, there were no alignments with weights between 0.67 and 1 in the no-iteration dataset, whereas the one-iteration MultiMap dataset had a much broader spectrum of possible weights.

Second, MultiMap improved the quality of mapping as measured by the analyses in Figures 3I-J. The curve of the proportion of correctly-positioned alignments for each weight was erratic in the no-iteration dataset and showed large deviations from linearity; while the one-iteration MultiMap dataset did also show some deviation from linearity, the curve was much smoother and closer to the diagonal than for its no-iteration counterpart (Fig 3I). Similarly, both the curves of the overlap proportion by alignment weight were highly linear, with the one-iteration MultiMap curve having slightly fewer deviations from linearity (Fig 3J).

Overall, these data suggest that the weights yielded from reweighting with MultiMap show improved concordance between alignment weight and the accuracy of the alignment, as measured by coincidence of alignments and overlap proportions. Thank you for suggesting this analysis – we find the results very exciting, and we think it offers a very useful validation of the MultiMap weighting scheme!

**2.4) Fig S2A is unclear. Why is the depth depending on number of iterations not just compared in a qq-plot against the golden standard depth as in S2B and S2C?**

Thank you – we should have been clearer on this matter. Our intention was for Figure S2A was not to show read depth or the distribution of read depth. Rather, we were attempting to convey the average mean absolute error for each iteration using our five-fold cross-validation method (conducted as per the figure legend).

However, upon reflection, we agree that the figure panel is confusing in etiology and in presentation, and we feel it offers no additional information of value. Accordingly, we have struck it from the figure and the manuscript.

As discussed in response to Reviewer #1 (Major Comment 1.1) and Reviewer #3 (Major Comment 3.6), we have instead replaced it with two analyses that better drive at why we see increased error with increased iteration – namely, analysis of mean error to assess for systematic skew in weights and slow reweighting to assess for random “overshoot” errors (Fig S2A-B).

**2.5) It might be just me, but I don't understand how the Golden Standard can have an off-target read depth (Fig 3G). Isn't Golden Standard the true depth at each locus?**

Thank you for bringing this up – it’s an important point on which we were insufficiently clear.

When we refer to “off-target” read depth in this context, we are referring to read depth found outside the set of pre-defined, randomly selected, “true origin” loci. The simulation tool we used (NEAT read simulator) takes this set of “true origin” loci as input and simulates Illumina reads such that there is roughly 30-fold coverage of reads over the “true origin” loci and roughly 6-fold coverage over genomic regions outside of these predefined loci. When we refer to off-target read depth, we meant to refer to the latter. We neglected to make this point in the manuscript; accordingly, to make this clearer, we have added language explaining this feature of the read simulator when the simulation is first introduced (lines 174-176).

**2.6) Fig 3J should probably be stratified by locus read depth. This way, one could see whether the multiMap approach generates some kind of biased errors towards high depth loci (see 1.).**

Good idea! To address this, we have conducted a mean absolute error analysis with the true origin loci stratified by Gold Standard read depth, normalized to Gold Standard read depth. This is presented in Fig S3F. First, we find that, as with the overall error estimates (Fig 3H and S3F), the MultiMap analyses outperform the corresponding uniread analyses and that the scored analyses outperform the corresponding unscored analyses (Fig S3F).

In addition, looking at all true origin loci overall, the one-iteration analyses yield lower mean absolute errors than do the no-iteration analyses (Fig 3H and S3F), and this relationship is preserved for loci with higher read depth (Fig S3F). Interestingly, however, for loci of lower read depth (particularly those with an average depth of less than 40), the no-iteration MultiMap analysis has a lower associated error than does the corresponding one-iteration MultiMap analysis. The reason for this is unclear. For all read depth categories, the mean error (rather than mean absolute error) is more negative for the one-iteration MultiMap than the no-iteration MultiMap analysis (Fig S3G). As such, it doesn’t appear that the reason for these differences is due to changes in systematic inflation of weights across loci of different read depths – at least, not to a significant enough degree that it is detectable by mean error analysis.

Whatever the reason may be for these differences, we feel it’s important to properly contextualize them. Both the no-iteration and one-iteration MultiMap analyses *considerably* outperform the uniread analyses in terms of mean absolute error (Fig 3H and S3F), and the differences between these two modalities of MultiMap are quite small for all of the different categories of locus depth. Nonetheless, we agree that it’s an interesting and useful analysis – thank you for the suggestion!

**2.7) For the sake of reproducibility, the authors should provide their data analysis code along with the paper, e.g. by citing a Zenodo archive or a git repository. Ideally, the analysis would be implemented in a fully reproducible, automated way (e.g. using a workflow management system).**

Good point! We have made the R scripts used for the analysis of the different methods available at <https://www.github.com/shah-rohan/multimap-analysis>. This provides the tools we used to compare different MultiMap or uniread modalities to each other for the simulated and biological datasets, as well as to the Gold Standard reference datasets in the case of the simulated data. This repository has also been updated to reflect the R code required for analyses in the responses to this review, as well as the tool that we used to generate our randomly sampled alignments for direct comparison to our MultiMap method.

**2.8) The golden standard dataset was generated with 50bp read length. Given current habits, this is an artificially low length, which probably artificially magnifies the advantage of multiread compared to uniread analysis. I think, to get a more up to date picture, the golden standard analysis should be repeated with 100bp reads.**

This is an excellent point. We have now conducted an additional Gold Standard analysis with simulated paired end 100bp reads, as presented in Figure S4 and Tables 1-2.

We find that despite the doubling in read lengths, MultiMap still considerably improves recovery of read depth and decreases the observed error to nearly the same extent as with 50bp reads. Notably, as per Table 1, the proportion of reads that are not uniquely mappable is roughly quite similar between the simulated datasets with 50bp reads and 100bp reads, and per Table 2, the proportion of regions gaining read depth between uniread and MultiMap analyses is also quite similar. Accordingly, when looking at the 100bp dataset, we see a largely similar distribution of alignments and reads by the number of alignments per read (Fig S4A-B), percentile plot of read depth (Fig S4C), QQ plots (Fig S4D-F), and mean absolute error (Fig S4G-H) as observed for the 50bp dataset (Fig 2-3, S3).

In these analyses, to be maximally conservative as to the benefit of MultiMap for 100bp reads and for more facile comparison to other figures, we have used the UMAP50 score (which is most appropriate for measuring mappability by 50mers) rather than the UMAP100 score, which measures mappability by 100mers; even so, at regions with low UMAP50 mappability scores, we *still* observe considerable improvement in read recovery and increased depth.

As such, we conclude that consideration of multireads is of nearly undiminished importance in the 100bp read length condition and that MultiMap can still increase read recovery and decrease error compared to the uniread condition. Thank you for the suggestion!

**2.9) The MultiMap software on Github definitely needs a Github release with proper versioning. Ideally, it should also be made available via Bioconda (but that is just a suggestion as I am biased in this regard).**

You are absolutely correct, thank you! We have created a Github release of the software and associated scripts as they were prior to review (v. 0.1.0) and as we have modified it for this review (v. 0.2.0). Thank you for pointing this out.

We feel that at this *particular* moment, it may be somewhat premature for us to make this software available via Bioconda – we would like to first ensure that our work is fully vetted by the full peer review process and that our responses to all of your concerns are satisfactory. We didn’t wish to consume the Bioconda reviewers’ time until this process has concluded but fully intend to submit this software after full review.

**2.10) The authors say that their reweighting is a Bayesian approach. I can see from the formulas that calculations resemble Bayes theorem, however the entire presentation is too ad hoc. The method presentation definitely needs an accurate definition of involved observed and latent variables, their modeled distributions and an explicit definition of their conditional dependencies. If the obtained weights are meant to be probabilities, please also define them as such, using the usual notation.**

Thank you for your comment; you are absolutely correct in that our presentation would benefit greatly from more rigor. To that end, we have rewritten the presentation of our algorithm to better describe the variables and their treatment in our method. Because our method relies upon Bayes’ theorem for its reweighting step (as more clearly outlined in this version), we feel it is still accurate to refer to our method as a Bayesian approach. However, if you feel another term would be more appropriate (for example, Reviewer #1 referred to our method as an EM method), then we would of course be happy to change it in the interests of accuracy.

**# Minor comments**

**2.11) Fig 3: the red and the orange are hard to distinguish.**

Thank you– we’ve fixed this.

**2.12) Fig 3E: How is excess read depth defined?**

Thanks for catching this. We define excess read depth as the difference between the MultiMap and uniread read depths; however, we originally failed to define this in the manuscript and have now done so (lines 272-273).

**2.13) Fig S2F: why are there gaps in the curve for M1S?**

Sorry for the confusion; because the curves for M1S and M1U were overlapping, we presented them as dashed lines so both could be seen. We have now made M1S a solid line so this is clearer and added a note to this point to the figure captions for Figure 3, S3, and S5.

**2.14) The performance comparison against the other method at line 554 seems quite selective (chosing just one method) and does not even run both methods on the same machine. To really make a statement about performance, this has to be done in a comparable setting on the same dataset. If the authors claim that their tool is performing best, the have to show it properly comparing against all others, on the same machine and data.**

Thanks for the suggestion. Our manuscript now includes a more detailed benchmarking table as Table 8, comparing our method to others with more detailed descriptions of the time and memory required for each step, as also requested by Reviewer #1 (Major Comment 1.2). As noted in response to your Major Comment #2, we were unable to completely compare MultiMap to BM-Map; we have noted that in our description of the computational efficiency of the method on lines 744-749. **Johannes Köster**

**Reviewer #3: In this paper the authors present a new approach and software for reweighting ambiguously mapped reads in NGS data, including ChIP-seq, ATAC-seq, and RNA-seq. Their approach uses binary indexed trees to store map counts and use an iterative algorithm to reweight alignments based on quality scores.

The primary application of this approach appears to be for “peak-type” NGS data, such as ChIP-seq and ATAC-seq, rather than RNA-seq. MultiMap does not directly output count data and thus does not easily fit into standard RNA-seq workflows. Also, reported challenges with gapped/spliced alignments would also disqualify MultiMap from RNA-seq analysis. However, the emphasis on peak data is actually an advantage for MultiMap since the ambiguous mapping problem in peak data has received far less attention than in RNA-seq (more on this below). Given the number of RNA-seq disambiguation approaches that have been proposed to date, the authors may consider focusing their tool on peak data and downplaying (or even removing) the RNA-seq applications.

The most novel aspect of their approach is that there is no need for a priori assumptions about genomic features of interest (lines 99-102). This sets MultiMap apart from every RNA-seq disambiguation approach. I think further emphasis of this point would help to distinguish their approach from existing approaches.**

These are both very interesting points, and we agree entirely – our work is more applicable for “peak-type” NGS data than for discontinuous data with predefined features such as RNA-seq. While we would still like to keep the data for the RNA-seq in the supplementary figure (in no small part to show data demonstrating that this method is not particularly useful for RNA-seq), we have worked to deemphasize the role of RNA-seq in MultiMap, including striking references to RNA-seq from the abstract and more clearly outlining in the results section that this method is not meant for RNA-seq. We hope that this better represents the best use case for MultiMap!

**A major weakness of this paper is that authors do not seem to be current on the relevant literature. The past 5 years has seen an explosion of new approaches for solving the problem of ambiguously mapped reads in RNA-seq. The review article by Lanciano and Cristofari (doi: 10.1038/s41576-020-0251-y) lists over a dozen approaches with this specific purpose.

Specific comments:

3.1) A thorough literature search would properly frame the importance and novelty of this work.
a. Line 96 - Should cite the review by Lanciano and Cristofari and the approaches therein.
b. Line 157 - There are previous works that use paired-end sequencing information and alignment quality.**

These are both good points – thank you for the suggested review! To the first point, we have now added the relevant information regarding the review by Lanciano and Cristofari at line 97.

To address the second point, you’re correct, and we should have been more careful in making that statement. Our intent was to communicate that no previous method had used the computationally efficient Fenwick tree structure for this kind of algorithm for paired-end sequencing reads, but we failed to communicate that initially. Instead, we have modified that paragraph to 1) correctly point out that other methods for “peak type” NGS analysis (most notably BM-Map) use paired-end reads with alignment quality and 2) to point out that this work expands upon the work of Chung et al. to use binary indexed trees for paired-end sequencing data (as you rightly note in Comment 3.5).

After re-scanning the literature on this topic, we were unable to find more methods applicable for our use case that had not been otherwise described.

Thank you for pointing this out!

**3.2) This study is missing a rigorous performance comparison with existing methods – the only method compared was unireads. If MultiMap is intended to be applied to RNA-seq data, it needs to be compared to RNA-seq methods (see Lanciano and Cristofari).**

**3.3) This study makes claims of improved computational efficiency but does not compare to existing approaches. Unireads approach (discarding ambiguous reads) will certainly be more computationally efficient.**

These are both great points, thank you!

As mentioned in response to similar concerns from Reviewers #1 and #2, we have now included a more detailed benchmarking table as Table 8, which outlines the computational requirements for MultiMap (with no reweighting and one reweighting iteration), uniread, and random alignment selection methods. Given the very valid points you raised about de-emphasizing the role for MultiMap in RNA-seq analysis, we have removed the strand-specific analysis from the benchmarking table and have thus not compared to RNA-seq methods. This table also includes mean absolute error as compared to Gold Standard for performance comparison between these different analysis methods.

As noted to Reviewer #2 (Major Comment 2.2), the other published methods that could be best compared to our work (as global “peak type” analyses rather than analyses of pre-defined regions) were CSEM and BM-Map. Unfortunately, these are both relatively old programs and may have been broken by changes to the language or the requisite libraries since their publication roughly a decade ago; CSEM returned an immediate segmentation fault, and BM-Map returned an error at Step 2 of the algorithm, after all the reads were read into the program memory. As such, comparisons of accuracy or computational efficiency to these works are challenging – though the very fact that these softwares do not function would argue in favor of developing new tools with modern libraries. Nonetheless, Table 8 includes the benchmarking comparisons possible from BM-Map to compare to MultiMap.

As noted to Reviewer #2, the other cited methods are either not particularly applicable to the kind of analysis (without external reference datasets) being done in MultiMap, so we have not compared to them – indeed, the very staging of such a comparison would be difficult, as described more completely in the response to Reviewer #2.

**3.4) I am not convinced that 51 alignments per read is sufficient. Some Alu families number in the tens of thousands of insertions and (depending on read length) may map hundreds of times. The peak at 51 in Fig 2 suggests that some reads have many more alignments and -k is too low.**

We agree entirely; we have no doubt that there are many reads that will map hundreds of times or more. And we completely agree that the peak at 51 suggests that some reads have many more alignments. Indeed, to that end, we do not analyze those reads mapping to 51 loci precisely because we cannot know for sure that they do not have more possible alignments.

The -k maximum alignment reporting value of 51 was chosen for computational feasibility; as the Bowtie2 manual suggests, reporting all alignments for a given dataset represents a significant computational challenge. To that end, we found that asking the aligner to report all alignments is extremely slow and outputs prohibitively large files to the point that our systems were unable to even completely align a dataset in that manner, let alone analyze it.

However, a few facts give us some amount of comfort here. First, the proportion of reads that have 51 reported alignments typically only comprise 2-5% of the total reads from sequencing (Table 1). Second, even with this limitation of only looking at reads with 1 to 50 alignments, we are still able to achieve good coverage over many repetitive elements, including Alu elements (Fig 6).

Third, and most importantly, this limitation is not an inherent problem of MultiMap; it is an alignment problem. In other words, if an end user is able to align their reads to as many loci as they desire, then there will not be anything stopping them from using MultiMap on the results (as long as their system is able to handle the computational load implicit in handling many more alignments and reads). The MultiMap algorithm is not restricted to use only up to 51 alignments; any maximum number of alignments can be used.

To this end, we have conducted analysis of our simulated dataset with a -k value of 101 such that up to 101 alignments will be reported. We find that much like the standard analysis, there are still many reads that align to at least 101 loci (Fig S5A-B), with the proportion increase in analyzable reads being similar to the analysis with max 51 reported alignments (Table 1). Similarly, the proportion of genomic windows with increased depth or with depth only by MultiMap relative to uniread is similar between the two alignment modalities (Table 2). Gains in read depth at true origin loci are also highly similar between the -k 51 and -k 101 datasets (Fig S5C-D), with a similar decrease in mean absolute error (Fig S5E-F). Finally, at repetitive element bodies, the read depths of the -k 51 and -k 101 datasets are highly similar and, indeed, very close to that of the Gold Standard (Fig S5G-J). As such, we think it’s fair to say that despite the fact that setting the max reported reads lower by default will exclude some regions from analysis, the impact of such a restriction is not too deleterious for many applications – and if needed, there’s nothing inherently limiting about MultiMap to prevent the end user from increasing the limit.

Nonetheless, we do agree that this is a limitation of our method that the manuscript should have been clearer on. The MultiMap method will only be as good as the quality of the alignment, and if the alignment has limitations, then those limitations will not be inherently fixed by MultiMap. To that end, we have added language to the manuscript making this point clearer (lines 796-812). Thank you for prompting us to do so!

**3.5) MultiMap algorithms, including Fenwick trees and iterative model, seem to be the same as presented in Chung et al. (doi: 10.1371/journal.pcbi.1002111). The main improvements appear to be support for paired reads and allowing for non-fixed number of iterations.**

We agree and feel that this is a fair assessment of how our work builds on previous studies. To be even fairer to Chung et al., their algorithm does not inherently forbid the use of a non-fixed number of iterations; they simply did not report using fewer than 200 iterations. However, as you correctly point out, their implementation of Fenwick trees and iteration did not support use of paired end reads, which we feel is one of the major features that we can offer with our study. **3.6) “Overfitting” may not be the proper term to describe deviation from the Gold Standard after the first iteration, since you are not adding parameters to the model. It is also interesting that your algorithm converges after a single iteration – it suggests that your model places too much weight on the (error-prone) observed data which may be incorrect due to sequencing error or mismatches with the reference. One way to mitigate this is to use an informative prior that assigns initial non-zero weights that are reweighted by the observed data.**

First, thank you for pointing out the poor use of “overfitting.” We have now changed this throughout the manuscript to “over-refining,” as that it a better description of what our method does – it refines the weights of the alignments iteratively.

We agree that it’s quite interesting that our algorithm converges after just one iteration, and we agree that this suggests that further iterations may place too much weight on error rather than the true trend.

However, it’s difficult to *a priori* think of an informative prior that could be generally applied to datasets, especially as external datasets can also suffer from problems of alignment and experimental differences. Instead, to address this point, we have implemented a new “slow reweighting” mode for our software. Previously, our algorithm would compute a new weight based on our Bayesian definition, determine the difference from the old weight, and change the weight of the alignment by that difference. This new “slow reweighting” method instead allows each iteration to only change the weight by a fraction of the difference between the theoretical new weight and the old weight.

For example, suppose the old weight of an alignment was 0.4 and the Bayesian definition of the new weight indicated that the new weight should be 0.8. Under the previous method, the weight of the alignment would be incremented by 0.4. However, our new “slow reweighting” method introduces a rate parameter *r* such that the weight will only be incremented by 0.4*r*. In this way, though we have not implemented an entirely novel informative prior, we have introduced a new way to more slowly fit the data to assess for convergence onto a set of weights.

To assess for this, we ran eight iterations of the MultiMap algorithm on the simulated dataset (with 50bp reads) using a weighting rate parameter *r* = 0.25 such that, in principle, eight iterations with *r* = 0.25 would result in roughly as much reweighting as two iterations of the previous type of MultiMap (where *r* = 1). We then computed the mean absolute error of the true origin loci as compared to the Gold Standard.

Interestingly, we found that the slow reweighting method yielded much more stable error estimates than did the normal MultiMap method. Rather than displaying the “check-mark” shaped curve seen in Figure 2D with standard MultiMap (*r* = 1), we found that with *r* = 0.25, the mean absolute error still was minimized with only one iteration, but the mean absolute error did not increase appreciably with further iterations (Fig S2B). Though the standard MultiMap yielded a slightly lower mean absolute error than did slow reweighting, the difference in error was small.

What this suggests to us is that the standard MultiMap algorithm may suffer from being overly reliant on noise and may “overshoot” the true weights with the reweighting, which will compound in error rapidly as further iterations are implemented. However, this can be mitigated by implementing a slower reweighting scheme as needed, which may have less of a risk of “overshooting” the true weights and will thus yield an error estimate that is more robust to iteration number.

Because standard and slow MultiMap are comparable in error after one iteration, we have left both methods as options in the software for maximum flexibility.

Thank you for this suggestion – we think that this new slow reweighting mode of MultiMap offers a very useful option for researchers and that it helps shed light onto how some of the problems with standard MultiMap can be avoided!  **3.7) Line 384: “impute” is used incorrectly**

Thank you for catching this – we have replaced this word with “utilize.”
