# Supplementary material for: Sequence deeper without sequencing more: Bayesian resolution of ambiguously mapped reads
Source: PLoS Comput Biol. 2021 Apr 19;17(4):e1008926. doi: 10.1371/journal.pcbi.1008926 (PMC8084338; doi:10.1371/journal.pcbi.1008926)
Supplement: Attachment — Submitted filename: Shah and Ruthenburg Response to Reviewer Comments 2.docx [file pcbi.1008926.s016.docx]

**Response to Reviewer Comments (Second Revisions)**

Thank you once again to all of the reviewers for reviewing our manuscript and evaluating our revisions to the original work. We are pleased to see that many of your concerns were addressed, and we really appreciate all your help!

Below, we have written a response to the remaining points and areas of concern. All line numbers refer to the clean version of the document.

**Reviewer #1: This revision has addressed my comments. While there are places where I think the language could use work ("as the old saying goes" is a tad colloquial for a technical paper but I defer to the editor :-)), and some of the figures look blurry to me, I think the reversion is overall extremely thorough and the paper is an exciting contribution.**

Thank you for your comments, and we are glad to hear that the revision has addressed your concerns! Upon reflection, we agree that such colloquialism isn’t the best for this kind of work and have struck that line from the manuscript. We believe the figures appear blurry because of the compression applied by the submission system – we will certainly ensure any figures submitted for the final work are of high quality and are clear! All the figures were made with vector graphics, so higher resolution is easily achieved if needed.

Thank you!

**Reviewer #2: Most of my requests were properly adressed. Two points remain below.**

**\# Major comments
* 2.7: I am glad to see the scripts being online in the github repo. Similar to the software, this repo as well needs a release that is properly cited in the paper. Apart from that, the analysis is still not reproducible. The repository lacks a readme, that would explain me how to run the analysis. It seems to lack steps that obtain the necessary reference data, and simulate the reads. It lacks the definition of a software environment to use (e.g. a Conda environment or a container image).**

Thank you, this makes sense to us now! In the interests of providing as much data and detail as possible for reproduction, we have decided to instead move forward with putting together a Zenodo repository (as you suggested as an option in the previous round of revisions) with Bash scripts and R scripts enabling analysis of the data and datasets for this paper, including the simulated and the biological datasets. In addition, to enable better reproducibility, we have included much of the simulated datasets (within the confines of size restrictions) so that the end user can run these analyses on the files that we generated. Finally, we have included a nearly 3000-word README file, both in the Zenodo repository and as part of our Github site for this project at <https://shah-rohan.github.io/MultiMap/analysis.html> to walk through the steps needed to simulate the simulated data, download the correct files for the biological data, and process the data completely.

The Zenodo repository with the analysis scripts and much of the simulated data can be found at <https://zenodo.org/record/4586639>. Due to space limits, the simulated FASTQ files are in a separate Zenodo repository, which can be found at <https://zenodo.org/record/4584103>. These have both been published as version 0.1.0 with a durable DOI. These accession numbers are both referenced in the manuscript.

We do have to confess that we were initially somewhat skeptical as to how useful the process of putting together this workflow would be. However, in the process of going through the scripts, we actually discovered two labeling errors in the manuscript, which we have now corrected.

The first error was the description of the region analyzed as a promoter for the repetitive elements; the manuscript described this as being from -50bp to +50bp relative to the 5’ end, when it was actually 100bp on either side. We have updated the text of the manuscript appropriately (line 1142).

The second error was in the presentation of Figure 3J, which was added in response to your Major Comment 2.3, in which we showed the proportion of the alignment that overlapped with the true read origin as a function of weight. We discovered in the process of putting this workflow together that this was mislabeled; these alignment overlaps were multiplied by the weight. As such, this figure panel instead represents the proportion of the reads’ weights that are correctly localized due to a given alignment as a function of weight. In addition to fixing the labeling and description of this figure panel (lines 368-378), we have also added in the analysis of the proportion of alignment and true read origin overlap as a function of weight as the new Figure S3H, with appropriate description in the manuscript (lines 378-381).

Much like Figure 3J, this new analysis shows that one iteration of MultiMap reweighting slightly reduces deviations from a high degree of alignment/origin overlap, although both the no-iteration and one-iteration analyses perform quite well. Accordingly, we feel that the takeaways from both the previous analysis (Fig 3J) and this new analysis (Fig S3H) are highly similar.

We sincerely apologize for these mistakes, and we are incredibly glad to have been able to correct them at this stage. Thank you for pushing us to put together a more robust reproducibility workflow, and we hope that this fits what you were looking for!

**# Minor comments
* Comment on the authors response to comment 1.5: I've still found the wording "golden standard" in Figure S2.**

Great catch, we’ve now fixed this. Thank you! **Johannes Köster**

**Reviewer #3: The authors adequately addressed all concerns and the new manuscript is much improved. The decision to position this tool mainly for “peak” data effectively solved many of the problems with its applications to transcriptome data.

I agree, it is very interesting that one iteration still gives the lowest error, and even with the slow-reweighting algorithm. I would be curious whether situations exist where multiple iterations would lower the error, though not necessary for this study.

Minor suggestions

Line 75-76: It is widely accepted that repetitive elements comprise >50% of the human genome. Typical citations establishing this include Lander et al. 2001 (initial human genome sequence), Wheeler et al. 2013 (Dfam), etc.**

Thank you for the suggested citations! We have added these in. **Line 191: writ -> at**

We have changed this, thank you! **Line 811-812: It has less to do with “poor quality” alignment as it has to do with the repetitiveness of the genome. Remember plants i.e. maize can have up to 90% repetitive DNA. Different genomes may require different -k values for optimal tradeoff between usable and discarded reads.**

This is a really good point, thank you! We’ve now added language to that paragraph elaborating upon the possible need to tune the -k value depending on the repetitiveness of the genome at lines 819-829.
