# Supplementary material for: Sequence deeper without sequencing more: Bayesian resolution of ambiguously mapped reads
Source: PLoS Comput Biol. 2021 Apr 19;17(4):e1008926. doi: 10.1371/journal.pcbi.1008926 (PMC8084338; doi:10.1371/journal.pcbi.1008926)
Supplement: Attachment — Submitted filename: Shah and Ruthenburg Response to Reviewer Comments 3.docx [file pcbi.1008926.s017.docx]

Thank you once again to the reviewers for their very helpful comments! Below, we have responded to the remaining recommendations.

Reviewer #2: I am happy to see the analysis being described quite comprehensively, thanks a lot for making this effort. I am also glad to see that not only the future readers but even the authors themselves even had a benefit from this, by discovering bugs in the previous analysis.

Thank you very much! We are glad to hear that your concerns were addressed!

Reviewer #3: The authors have done a thorough job addressing the reproducibility concerns by including additional documentation and example data sets.

Suggestions:

Note that MultiMap is the name of an existing computational genetics software (PMID:8054979) with 109 citations.

That’s an excellent catch, thank you! To avoid confusion, we have renamed our software SmartMap, a name which does not appear to be used in the field. (Its only other instance in Pubmed is a little-cited geographic information service; we don’t anticipate any confusion with our software.)

I do think the point that Dr. Köster is making is valid – if your goal is to produce software that can be used by others, there is still more that can be done. When I see a new software that has 10 dependencies that need to be installed, I usually just look for another package. Best practices would be to provide a way that dependencies can be easily managed, and a package manger (like conda/bioconda) is one of the easiest ways to do this. With conda you simply list these dependencies in a YAML file and conda takes care of resolving them.

This is a great point, and it also lines up very well with a recommendation that Dr. Köster made in the first round of review to deposit our software with Bioconda. At that time, we indicated that we wanted to first go through rigorous peer review before distributing the tool more broadly; we now feel comfortable with making SmartMap more widely accessible and have accordingly created a conda package and deposited with Bioconda. The package can be found here: <http://bioconda.github.io/recipes/smartmap/README.html>, with standard installation instructions for a Bioconda package. Thank you for the recommendation!

Simulation data can be more easily made available by providing the simulator command and specifying a random seed. Since simulations are relatively inexpensive to generate but can easily produce terabytes of data, it is much more effective to provide simulation scripts that can precisely recreate your simulations. Most simulators will reproduce the exact simulated sequence by specifying a random seed; see “-seed” for bedtools and “--rng” for NEAT.

This is a great point, thank you. Unfortunately, it doesn’t seem to be feasible at this point to provide these seeds for this study – we did not set the seeds when we generated this data roughly a year ago, and accordingly can’t provide those as an alternative to the full data upload. For that reason, we’ve uploaded our simulation data to Zenodo instead to make it available for the end user in multiple formats and to reduce the computational burden on the end user to generate these datasets de novo. Nonetheless, we sincerely appreciate the suggestion, and for future studies, we will be sure to keep this in mind to enable more flexible reporting of reproducibility data!
